# Timing of Endotracheal Intubation in Patients with Fulminant Enterovirus 71 Infection

**DOI:** 10.3390/medicina56040203

**Published:** 2020-04-24

**Authors:** Shen-Dar Chen, Ying-Tzu Ju, Yu-Jen Wei, Min-Ling Hsieh, Ching-Chuan Liu, Jing-Ming Wu, Jieh-Neng Wang

**Affiliations:** 1 Department of Pediatrics, Dalin Tzu Chi hospital, Buddhist Tzu Chi Medical Foundation, Chia-Yi 62247, Taiwan; ericcdar@yahoo.com.tw; 2 Department of Pediatrics, National Cheng Kung University Hospital, College of Medicine, National Cheng Kung University, Tainan 70421, Taiwan; ints.ju@gmail.com (Y.-T.J.); garlicwei@hotmail.com (Y.-J.W.); mactep_8@hotmail.com (M.-L.H.); liucc@mail.ncku.edu.tw (C.-C.L.); jingming@mail.ncku.edu.tw (J.-M.W.)

**Keywords:** enterovirus 71, timing, intubation, emergency care

## Abstract

*Background and objective*: Enterovirus 71 (EV 71) infections may result in the rapid progression of cardiopulmonary failure. Early endotracheal intubation is considered to be of primary importance. However, the appropriate timing for this is still not known. The aim of this study is to investigate the timing of intubation of children with fulminant EV71 infection. *Material and Methods*: From March 1998 to May 2012, patients with severe EV71 infection who were admitted to the pediatric intensive care unit of the National Cheng Kung University Hospital were enrolled in this study. Medical records were retrospectively reviewed. The patients were classified into three groups in accordance with the outcome of intubation. We used rhombencephalitis grading to describe the neurological presentation of these patients. The study was approved by the institutional review board. *Results*: There were a total of 105 patients enrolled. Of these, 77 patients were in Grade I, and only three of them needed intubation, who were, however, soon extubated within 24 h. There were 10 patients in Grade II; nine of them needed intubation. In total, 18 patients belonged to Grade III, and all of them need to be intubated. We then compared the outcome of intubation of grades II and III. There was only one patient out of the nine patients in grade II who experienced failed extubation due to the progression of the disease. Among grade III patients, only four patients were successfully extubated. We also listed clinical parameters to determine which one could be a sign that indicated intubation. Comparing the favorable outcomes, cranial nerve involvement was a good indicator for the timing of intubation. *Conclusions*: This study showed that early intubation in Grade II provides favorable outcomes and improves morbidity and mortality. We also found that if cranial nerve involvement was present, then early intubation is indicated.

## 1. Introduction

Enterovirus 71 (EV 71) infections may result in severe complications such as neurological sequalae, cardiopulmonary failure, and death [1,2,3,4]. Most of the fatal cases initially involved minor neurological symptoms (myoclonic jerk, oculomotor paresis, etc.), but the children rapidly developed pulmonary edema (PE), pulmonary hemorrhage (PH), and rapid progression of cardiopulmonary failure within hours after admission [3,4,5,6]. The progression of this disease is difficult to predict. Much discussion has taken place regarding clinical staging and critical care management [7,8,9,10,11,12,13]. For patients with progressive deterioration, early endotracheal intubation (ETI) for impending cardiopulmonary failure and providing the best supportive and respiratory care are considered to be of primary importance. ETI could prevent unexpected hypoxia due to CNS complications which might produce secondary brain injury. In addition, early ETI may also protect further injury to the central nervous system (CNS), especially the brain stem, which is the important of airway behavior center [14], and ETI may produce a favorable outcome for these patients. However, it is still not known when the appropriate timing is to perform ETI in these patients. The outcome of early intubation has also not yet been reviewed. Therefore, our objective was to report our experiences about when the appropriate timing for ETI is and whether early intubation may improve the outcome of fulminant EV71 patients.

## 2. Materials and Methods

We retrospectively reviewed the medical records of severe EV71-infected children who were admitted to the pediatric intensive care unit (PICU) in the National Cheng Kung University Hospital, from March of 1998 to May of 2012. Those patients were admitted to PICU because of the progression of the disease, and frequent hemodynamic monitoring was required. The study protocol was approved by our Institutional Review Board on 13 November 2019 (A-ER-108-419).

Enterovirus infection was defined by the isolation and typing of EV71 from rectal or throat swabs, cerebral spinal fluid, or increasing antibodies by using the neutralization method. The definitions of EV71’s clinical stages and rhombencephalitis grades were according to the World Health Organization (WHO) guidelines [15] and Huang CC, et al. [9] (Table 1). We reviewed the clinical presentations and carefully examined the reasons for ETI in each subject. In our hospital, endotracheal intubations were performed by intensivists or senior pediatric residents (intensive care trained) in PICU. We used the oral approach in every cases. As soon as the need for intubation was recognized, pre-oxygenation with 100% oxygen for 2–5 min was given. Lidocaine (1.5 mg/kg) was used for pre-medication to blunt the intracranial pressure response to intubation, midazolam (0.1–0.3 mg/kg) for sedation, and rocuronium (0.6 mg/kg) for muscle paralysis. We did not use atropine because the patients with fulminant enterovirus 71 infection had extreme tachycardia already. Appropriate endotracheal tube size and laryngoscope were used according to the patient’s size. Appropriate ET tube placement was verified with auscultation, observation for symmetrical chest rise, capnometry, and chest radiography. The classifications of the clinical stage and rhombencephalitis grade were according to the medical record, and we evaluated and recorded the clinical stage or rhombencephalitis grade during the PICU admission period in each patient. Early intubation was defined as intubation under stable hemodynamics without shock status and a standard rapid sequence induction procedure. The ETI was considered unnecessary if the ETI duration was less than 24 h. The definition of cranial nerve involvement included ocular disturbances (nystagmus, strabismus, or gaze paresis) and bulbar palsy (dysphagia, dysarthria, dysphonia, and facial weakness) [9]. The definition of hypertension and hypotension were age-dependent and based on values published by the Task Force on Blood Pressure Control in Children [16]. The patient management was standardized and based on treatment protocol after 2002 [10]. Intravenous immunoglobulin (IVIG) with a dosage of 1–2 g/kg was given if the disease was progressed or presented with clinical stage II or rhombencephalitis grade II and above. The clinical outcome was also recorded. The definition of poor outcome was death, ventilator-dependence, or a vegetative state with major neurological sequelae. Fisher’s exact test was used to compare the clinical significance, which was determined at *p* < 0.05. A one-way ANOVA test was used to compared age and oxygen saturation among groups.

## 3. Results

In total, 105 patients were enrolled in this study. The age of the patients ranged from 2 months to 5.5 years. The gender ratio of males to females was 1 over 1.1. We found that most of the patients received intubation owing to the deterioration of vital signs such as persistent tachycardia (86.7%), frequent myoclonus (73.3%), irregular respiration (60.0%), and development of shock (46.7%). The heart rate, blood pressure, and oxygen saturation were not significantly different between the non-intubated patients versus intubated patients. However, patients with poor outcome presented with significant younger age, poor hemodynamics status (heart rate, blood pressure), and irregular respiration status. Four patients in the poor outcome group presented with hypertension initially but soon deteriorated into hypotension during the preparation of intubation. The comparison of clinical presentations in 71 intubated or non-intubated enterovirus patients and their related outcomes is shown in Table 2.

In the WHO clinical staging classification, there were 43, 45 and 17 patients categorized into stage II, III and IV, respectively. However, by using rhombencephalitis grading, 77 patients were categorized into Grade I, 10 patients into Grade II and 18 patients into Grade III, respectively. We compared the clinical staging and rhombencephalitis grading in these patients. By comparing these two classifications, the patients who were in clinical staging II and rhombencephalitis grade I shared the same feature, i.e., that intubation was unnecessary. On the other hand, nearly all patients in stage IV and rhombencephalitis grade III needed to receive ETI. Therefore, differences existed between clinical stage III and rhombencephalitis grade II (Table 3).

We found that in the clinical staging group, stage III, only 28.9% patients received intubation compared to rhombencephalitis grade II, in which 90% of patients needed intubation. When we compared the outcomes, patients who were intubated in grade II had the highest predictive good outcomes versus grade III (Table 3).

## 4. Discussion

This is the first study to address the timing of ETI in the management of severe EV71 infection. According to our data, we found that using rhombencephalitis grading was very sensitive in the prediction of the need of ETI and its relation to good outcomes compared to clinical staging.

EV71 infections may invade the brainstem of patients and rapidly progress to pulmonary edema or pulmonary hemorrhage. The pathogenesis remains unknown. Some have hypothesized that brainstem involvement may induce autonomic nervous system dysregulation, tachycardia, and rapid changes of the vascular tone and resistance and pulmonary edema [3,7,17]. As we know, the brainstem is the area in which cranial nerves arise and the respiratory center. We can postulate that the progression of these patients may present cranial nerve involvement, leading to cardiopulmonary failure. Early intubation may improve the oxygenation of the brainstem and may prevent further insults. In this study, our results showed that nine of the ten patients in rhombencephalitis grade II received early intubation and the outcomes were good. 

The timing of intubation for impending cardiopulmonary failure patients is controversial. Delayed intubation is associated with increased mortality in moderately traumatic injured patients [18]. Severe studies would suggest that some pediatric patients suffering from respiratory insufficiency with appropriate mental status and airway reflexes may benefit from noninvasive ventilation and potentially be able to avoid or delay endotracheal intubation and mechanical ventilation [19,20,21,22,23,24,25]. Due to limited case experiences, we did not find benefits of early noninvasive ventilation for fulminant EV71 infection due to the rapid progression of cardiopulmonary failure. The use of noninvasive ventilation in these patients might require further research. According to current WHO guidelines [14], it is suggested to consider early intubation only during the autonomic nervous system (ANS) dysregulation stage (stage III), but there was no specific recommendation for the timing of intubation. Our data showed that symptoms and signs of cranial nerve involvement might be good indicators for early intubation. 

The limitation of this study was this was not a randomized prospective study. The results may be biased because we only compared patients who were intubated. The large scale of the sample may require more persuasive data. Besides, there was no comparison data between non-intubation and intubation patients in grade II. Only one patient in grade II did not receive ETI. Moreover, the severity of the disease was not revised. The patient outcome improvement may have been due to the less severe nature of grade II patients if compared to grade III patients. However, there were two patients who progressed to grade III without intubation. When these patients progressed to grade III, the prognosis was worst. The question of whether every patient in EV71 infection will present with cranial nerve involvement before progressing to cardiorespiratory failure has still not been solved. It was very difficult to predict the progression of the disease. However, we may identify these patients if they were under close surveillance in an intensive care unit.

## 5. Conclusions

EV71 infection is a highly contagious disease and may cause severe morbidity and mortality to young children. Clinical staging can in practice describe the progression of the disease, but it does not show much benefit in terms of the timing of intubation and outcome prediction. We found that early intubation is indicated for brainstem protection. Rhombencephalitis grading is more suitable for the timing of intubation and may improve the outcome of EV71 patients.

## Figures and Tables

**Table 1:** Comparison of enterovirus 71 (EV71) infection clinical stages and rhombencephalitis grades.

	World Health Organization (WHO) Guidelines *	Rhombencephalitis Grading **
**Stage I:**	Uncomplicated hand, foot, and mouth disease (HFMD)/Herpangina Stage	
**Stage II:**	HFMD with Central Nervous System (CNS) Involvement Stage	**Grade I:** Myoclonus with tremor, ataxia, or both
	Aseptic meningitis/Brainstem encephalitis/Encephalomyelitis	
	Most patients present with subtle neurologic symptoms or signs (e.g., drowsiness, limb weakness, ataxia, and myoclonic jerks)	
**Stage III:**	HFMD with Autonomic Nervous System (ANS) Dysregulation Stage	**Grade II:** Myoclonus with cranial-nerve involvement
	Tachycardia, hypertension, profound sweating, respiratory abnormalities	
**Stage IV:**	HFMD with Cardiopulmonary Failure Stage	**Grade III:** Rapid cardiopulmonary failure
	Hypotension/Shock, pulmonary edema/hemorrhage	

* by WHO, Reference [14]. ** by Huang CC, et al., Reference [9].

**Table 2:** Comparison of demographic data and clinical presentations in intubated or non-intubated enterovirus 71 patients and their related outcome.

	Total Patient Number (*n* = 105)	Non-Intubated Patient Number (*n* = 75)	Intubation Patient Number (*n* = 30)	Poor Outcome * (*n* = 14)
Age (months)	30.7 ± 20.3	38.1 ± 22.2	23.5 ± 17.7	15.9 ± 12.7 ***
Oxygen saturation (SpO2) (%)	95.5 ± 5.1	96.5 ± 4.8	93.2 ± 7.8	91.4 ± 6.1
Myoclonus	85 (81.0%)	63 (84.0%)	22 (73.3%)	11 (78.5%)
Seizure	21 (20.0%)	14 (18.7%)	7 (23.3%)	3 (21.4%)
Irregular respiration	19 (18.1%)	1 (1.3%)	18 (60.0%)	12 (85.7%) ***
Ataxia	20 (19.0%)	14 (18.7%)	6 (20.0%)	2 (14.3%)
Cranial nerve involvement **	12 (11.4%)	1 (1.3%)	11 (36.6%) ***	0 (0%)
Tachycardia	58 (55.2%)	32 (42.7%)	26 (86.7%) ***	12 (85.7%)
Hypertension	34 (32.4%)	23 (30.7%)	11 (36.7%)	4 (28.6%)
Hypotension/Shock	14 (13.3%)	0 (0%)	14 (46.7%)	14 (100%) ***

* Poor outcome including death, ventilator-dependent, or vegetative with major neurological sequelae. ** Cranial nerve involvement including ocular disturbances (nystagmus, strabismus, or gaze paresis) and bulbar palsy (dysphagia, dysarthria, dysphonia, and facial weakness). *** *p* < 0.05 by Fisher’s exact test or one-way ANOVA test.

**Table 3:** Comparison of intubation outcome by using rhombencephalitis grading and clinical staging in severe EV71 patients.

Clinical Staging	Number of Patients	Number of Patients Received ETI (%)	Poor Outcome *	Rhombencephalitis Grading	Number of Patients	Number of Patients Received ETI (%)	Poor Outcome *
II	43	1 (2.3%)	0 (0%)	I	77	3 (3.9%)	0 (0%)
III	45	13 (28.9%)	0 (0%)	II	10	9 (90%) **	0 (0%)
IV	17	17 (100%) **	14 (87.5%) **	III	18	18 (100%) **	14 (83.3%) **

* Poor outcome including death, ventilator-dependence, or vegetative state with major neurological sequelae. ** *p* < 0.05 (by Fisher’s exact test).
